# Editorial: Immuno-oncologic biomarker signatures for personalized immunotherapy and immunoprevention in oral squamous cell carcinoma

**DOI:** 10.3389/froh.2025.1635675

**Published:** 2025-06-16

**Authors:** Rania H. Younis, Ranee Mehra, Nikolaos G. Nikitakis

**Affiliations:** ^1^ Department of Oral Pathology, Faculty of Dentistry, Alexandria University, Alexandria, Egypt; ^2^ OralPath-DentCare, LLC, Ellicott City, Maryland; ^3^ Department of Medicine, School of Medicine, University of Maryland, Baltimore, MD, United States; ^4^ Head and Neck Oncology, The University of Maryland Marlene and Stewart Greenebaum Comprehensive Cancer Center, Baltimore, MD, United States; ^5^ Department of Oral Medicine & Pathology and Hospital Dentistry, School of Dentistry, National and Kapodistrian University of Athens, Athens, Greece

**Keywords:** oral cancer, inflammation, stroma, fibrosis, prevention, personalized, immunotherapy

**Editorial on the Research Topic**
Immuno-oncologic biomarker signatures for personalized immunotherapy and immunoprevention in oral squamous cell carcinoma

The development of oral cancer is a multifactorial and complex process (1). The interaction between the mutated oral epithelial cells and the underlying stromal and immune system plays a major role in tumor development and progression. Immunotherpay is one of the most recent therapeutic advents in the field and has significantly improved overall survival. However, the differential responses of the patients warrant a better understanding of the immuno-oncologic profile of oral cancer.

The current research topic in *Frontiers in* *oral health—oral cancers* aimed to shed light on recent evidence-based immuno-oncological biomarkers associated with the clinicopathological features of oral squamous cell carcinoma (OSCC) and potentially cancerous oral lesions. The ultimate goal is for better patient stratification and more guidance for personalized immuno-therapy regimens (2).

The research topic starts with a contribution by Sahu et al., titled: *4-Nitroquinoline 1-oxide induces immune cells death to onset early immunosuppression during oral squamous cell carcinoma development.* In this manuscript, the authors presented an *in vivo* model showing how the chemical carcinogen modulates an immunosuppressive profile that starts in oral precancerous stages. A significant decrease in B and T cell populations, specifically the CD5 + B cell, and gamma/delta-T cell (γδ-TCR^+^) populations was observed in the spleen in the early stages of development of the precancerous lesions and persisted after OSCC development. Despite an increase in NK, NKT, and lymphoid DCs in the peripheral blood, there was a decrease in the majority of lymphocyte populations (T, B, CD5 + B cells) during the early stages of development, but not later on. The authors concluded that immune cell derangement is not a post-cancerous event, but rather an early event that may persist until tumor development and beyond. *In vitro* analysis demonstrated that B cells are more susceptible to reduced viability and DNA damage when exposed to the chemical carcinogen compared to T cells. These findings provide preclinical evidence supporting the introduction of immunotherapy in the initial stages of precancerous lesions, as a preventive strategy for OSCC.

To further highlight the onset of immune derangement early in OSCC precursor lesions, Hanroongsri et al., in *Expression of PD-L1 and p-RPS6 in epithelial dysplasia and squamous cell carcinoma of the oral cavity*, aimed to describe the significant correlation between the expression of two well-established immunological and oncological biomarkers, programmed death ligand -1 (PD-L1) and phosphorylated ribosomal protein S6 (RPS6), respectively, in oral epithelial dysplasia (OED). In a cohort of 52 OSCC and 48 OED cases, p- RPS6 expression was detected in all cases, whereas PD-L1 was detected in 42/48 (87%) OED cases and 28/52 (53%) OSCC cases. Patients with mild OED presented a significantly higher expression of PD-L1 and p-RPS6, when compared to moderately differentiated OSCC patients (*P* < 0.05). The researchers described a significant positive correlation between PD-L1 and p-RPS6 expression in OED and OSCC patients. Their findings suggest that the upregulation of PD-L1 may be related to the activation of the mitogenic pathway mTOR in the early stages of precancerous lesions and in the pathogenesis of OSCC. Their work provides additional evidence supporting the idea that the process of immune modulation can occur concurrently with the mitogenic progression of precancerous and tumor cells.

In addition, the research topic includes a study by Theofilou et al., *Histological pattern of tumor inflammation and stromal density correlate with patient demographics and immuno-oncologic transcriptional profile in oral squamous cell carcinoma*. The authors examined 87 prospectively collected OSCC samples to evaluate the histological scoring of tumor inflammation and fibrosis. The transcriptional and KEGG pathway analyses included two to three pairs of each clinicopathological subtype. The study characterized an association between histologically inflamed tumors (HIS-INF) and female gender, individuals who do not drink, and small tumor size. Peritumoral stromal fibrosis (HIS-SF), on the other hand, was associated with the male gender, individuals who smoked, alcohol consumption and larger tumor size. The immune-excluded tumor (HIS-IE) subtype showed upregulation of the matrix metalloproteinases (MMP 7 and MMP9), and oncogenic pathways (PKC, PI3K, HIF-1β, and PPARG), with decreased cytokine signaling and dominated by myeloid cells. This work highlights the translational potential of characterizing the histological pattern of tumor inflammation and stromal density, which can be linked to oncological pathways to generate complex immuno-oncologic signatures for optimal patient stratification.

A systematic review by Ribeiro et al., *The prognostic significance of tertiary lymphoid structures in oral squamous cell carcinomas: a systematic review*, included six retrospective cohort studies involving 1,203 patients that met the inclusion criteria. The researchers aimed to explore tertiary lymphoid structures (TLS) in the tumor microenvironment (TME). TLS were found to be predominantly located in the peri-tumoral area (75.4%–84.8%) compared to the intra-tumoral area (33.8%–33.9%). The review showed that the presence of TLS is associated with improved survival in OSCC patients. They concluded that standardization of methodologies for detecting TLS is imperative to ensure consistency in criteria utilization, thereby facilitating meaningful data comparison. Their work adds to the importance of characterizing tumor inflammation and confirms the long-standing notion among pathologists that TLS is usually associated with low-grade tumors, a responsive immune system, or tumors with a better prognosis.

The fifth article, by Acharya et al., *Prognostic significance of IL-33 and ST2 expression in head and neck squamous cell carcinoma: a Systematic Review*, evaluated the upregulated expression of the inflammatory cytokine IL-33 in cancer-associated fibroblasts (CAFs) as an emerging immune biomarker of unfavorable clinical outcomes. Nine studies that met the inclusion criteria were examined. These described the abundance of IL-33-expressing CAFs associated with ST2 expression on regulatory T cells (Tregs). IL-33 primarily binds to the ST2 receptor expressed in immune cells, such as Tregs, suggesting that it can have pro- or anti tumorigenic effects, depending on the cellular context. The authors highlighted the IL-33/ST2 axis, which triggers NF-kB, as a significant driver of tumor growth. They concluded that IL-33 may be a prognostic biomarker and therapeutic target in HNSCC, which significantly configures the tumor microenvironment and tumor aggressiveness. They also discussed the role of serum IL-33 and ST2, which requires further study in HNSCC, as the soluble form of ST2 may function as a decoy receptor reducing IL-33 biologic activity. This highlights the implications of soluble vs. cell-bound inflammatory cytokines, which may have different biological and translational significance.

The five studies presented in the current research topic emphasized traditional, emerging and novel complex biomarker signatures of the immuno-oncologic platform. This area of research focus will help stratify OSCC patients and accordingly open new avenues for better personalized lines of treatment, early detection, and OSCC prevention.
